# In Silico Investigation of Selected Pesticides and Their Determination in Agricultural Products Using QuEChERS Methodology and HPLC-DAD

**DOI:** 10.3390/ijms24098003

**Published:** 2023-04-28

**Authors:** Stefan Petrović, Biljana Arsić, Ivana Zlatanović, Jelena Milićević, Sanja Glišić, Milan Mitić, Rada Đurović-Pejčev, Gordana Stojanović

**Affiliations:** 1 Department of Chemistry, Faculty of Sciences and Mathematics, University of Niš, Višegradska 33, 18106 Niš, Serbia; stefan.petrovic@pmf.edu.rs (S.P.); ivana.zlatanovic@pmf.edu.rs (I.Z.); milanmitic83@yahoo.com (M.M.); gordana.stojanovic@pmf.edu.rs (G.S.); 2 Laboratory for Bioinformatics and Computational Chemistry, Vinča Institute of Nuclear Sciences, The University of Belgrade, Mike Petrovića Alasa 12-14, Vinča, 11351 Belgrade, Serbia; sanja@vin.bg.ac.rs; 3 Institute of Pesticides and Environmental Protection, Banatska 31b, 11080 Belgrade, Serbia; rada.djurovic@pesting.org.rs

**Keywords:** agriculture, toxicity, Alzheimer’s disease, EIIP, *Mus musculus*, *Homo sapiens*

## Abstract

In this study, we considered some pesticides as active substances within formulations for the protection of plant-based food in the Republic of Serbia in silico, because these pesticides have not often been investigated in this way previously, and in an analytical way, because there are not very many available fast, cheap, and easy methods for their determination in real agricultural samples. Seven pesticides were detected in selected agricultural products (tomatoes, cucumbers, peppers, and grapes) using the QuEChERS methodology and HPLC-DAD. Standard curves for the investigated pesticides (chlorantraniliprole, methomyl, metalaxyl, thiacloprid, acetamiprid, emamectin benzoate, and cymoxanil) show good linearity, with R^2^ values from 0.9785 to 0.9996. The HPLC-DAD method is fast, and these pesticides can be determined in real spiked samples in less than 15 min. We further characterized the pesticides we found in food based on physicochemical properties and molecular descriptors to predict the absorption, distribution, metabolism, elimination, and toxicity (ADMET) of the compounds. We summarized the data supporting their effects on humans using various computational tools to determine their potential adverse effects. The results of our prediction study show that all of the selected pesticides considered in this study have good oral bioavailability, and those with high toxicity, therefore, could be harmful to human health. Chlorantraniliprole was shown in a molecular docking study as a good starting point for a new Alzheimer’s disease drug candidate.

## 1. Introduction

Pesticides are a broad class of chemical compounds used to control pests and pathogens. Despite the benefits of their use, their continued application and release into various ecosystems have become a problem for humans and the environment [1].

Carbamate pesticides are widely used to protect fruits and vegetables from insects, fungi, plant diseases, and weeds. Due to their good properties, such as broad biological activity, low bioaccumulation potential, low mammalian toxicity, and relatively short life span, they are popularly used in agriculture and are better substitutes for organochlorines and organophosphates. However, the negative side of carbamate pesticides is their harmful effect on the central nervous system, as they can bind to the active sites of the enzyme acetylcholinesterase (AChE) [2,3,4].

The complexity of real samples usually requires their preparation to eliminate the effects of the matrix and increase the concentration of the analyte (pesticide). In previous studies, the following pretreatment methods were used for the extraction and concentration of the desired pesticides: microwave-accelerated selective Soxhlet extraction (MA-SSE) [5], single-drop microextraction (SDME) [6], dynamic microwave-assisted extraction online coupled with single drop microextraction (DMAE-SDME) [7], dispersive solid-phase extraction (DSPE) [8], hollow fiber liquid-phase microextraction (HF-LPME) [9], magnetic solid-phase extraction (MSPE) [10], magnetic graphene solid-phase extractions (G-MNPs) [2], cloud-point extraction (CPE) [4], ultrasound-assisted surfactant-enhanced emulsification microextraction (UASEME) [11], quick easy cheap effective rugged and safe (QuEChERS) [12,13], and molecularly imprinted solid-phase extraction (MISPE) [14].

The standard QuEChERS method is usually subjected to minor or major modifications. Samples with complex matrices, i.e., samples with a high lipid content and highly pigmented samples, require additional purifications by introducing a solid phase with a highly active adsorption surface, such as C18 and charcoal [15]. Lehotay et al. (2005, 2010) [16,17] modified the standard QuEChERS method for the extraction of unstable pesticides from fruit and vegetable samples by adding buffers. They achieved the desired pH by adding anhydrous sodium acetate during the extraction of the pesticides from the fruit and vegetable samples.

Farina et al. (2017) [18] quantified 15 different pesticides in the leaves of leafy vegetables (cabbage, broccoli, cauliflower, lettuce, celery, spinach, and mustard). Another group of researchers conducted a study intending to determine pesticide residues in 120 vegetable samples (parsley, lettuce, and spinach) [19]. Andrade et al. (2015) [15] developed a very rapid method to quantify 57 pesticides in 58 tomato samples in only 13 min using liquid chromatography–electrospray ionization tandem mass spectrometry. They determined the following pesticides, among others: thiamethoxam, acetamiprid, cymoxanil, thiacloprid, and tebuconazole. Sinha et al. (2012) [13] improved the QuEChERS method for the extraction of 18 organophosphates from vegetable samples and quantified them using liquid chromatography–mass spectrometry. Among other carbamate pesticides, methomyl was also determined in the fruit samples. Moreno-González et al. (2013) [11] determined carbamate pesticides in wine samples, including methomyl and cymoxanil.

The aim of this research was the in silico investigation of selected pesticides and their determination in agricultural products using QuEChERS methodology and HPLC-DAD. Inhibition capabilities of selected pesticides in silico against acetylcholine esterase of *Mus muculus* and *Homo sapiens* were checked. In this regard, this research is the continuation of our previous study [20]. To the best of our knowledge, nobody before us has performed such an analysis giving insight into the different toxicities of pesticides against *M. musculus* and *H. sapiens*. As some pesticides have been shown to be reversible inhibitors of acetylcholine esterase, they are used in the treatment of myasthenia gravis, Alzheimer’s disease, postoperative ileus, bladder distention, and glaucoma, as well as as an antidote to anticholinergic overdose [21]. Three approved drugs (donepezil, rivastigmine and galantamine) were docked into the binding site of donepezil in acetylcholine esterase of *H. sapiens* and experimentally determined pesticides (chlorantraniliprole, methomyl, metalaxyl, thiacloprid, acetamiprid, emamectin benzoate, and cymoxanil), and their binding abilities were compared. Recently, we screened natural compounds for candidate 5HT6 receptor antagonists against Alzheimer’s disease [22].

The reason for using in silico approaches is the relatively lower cost and time factor compared to standard experimental approaches for ADMET profiling [23]. The amount and diversity of data obtained from experimental toxicity studies allowed the construction of truthful computational models for toxicological evaluation. They are recognized by the European Food Safety Association [24] and are nowadays constantly used in the evaluation of the toxicological effects of different pesticides on humans [25,26]. We use some of these computational tools in our study because they were developed for predicting the effects on humans. Therefore, early optimization in this process is very important.

## 2. Results and Discussion

### 2.1. Determination of the Contents of Selected Pesticides in Selected Agricultural Products

Standard curves for the investigated pesticides (chlorantraniliprole, methomyl, metalaxyl, thiacloprid, acetamiprid, emamectin benzoate, and cymoxanil) show good linearity (Table 1).

Thiacloprid, methomyl, metalaxyl, and chlorantraniliprole can be determined in less than 15 min in the tomato sample treated with the QuChERS kit for highly pigmented samples (P2) and without it (P1) (Figure S1). Retention times of selected pesticides are negligibly different in real spiked samples compared to standard solutions of the pesticides alone due to the matrix effect [27]. In P2, only thiacloprid was determined in the real sample without spiking. The concentration of chlorantraniliprole was 1.43 mg/kg, that of thiacloprid was 0.77 mg/kg, and methomyl was found to be present at a concentration of 0.85 mg/kg. Metalaxyl was not quantified because its quantity was below the limit of quantification. In sample P1, thiacloprid and metalaxyl could not be quantified, the concentration of methomyl was 0.96 mg/kg, and the concentration of chlorantraniliprole was 1.56 mg/kg. Thiacloprid ((*Z*)-3-(6-chloro-3-pyridylmethyl)-1,3-thiazolidin-2-ylidenecyanamide) is effective against sucking and biting insects [28]. A matrix effect was observed in the determination of thiacloprid in green tea [28]. The treated tea samples have concentrations in the range of 0.04–2.55 mg/kg (fresh) and 0.04–1.23 mg/kg (dry) [28]. The conclusion was drawn based on the research that elevated temperatures can lead to the degradation of thiacloprid, which has been observed previously [29]. The initial deposition of thiacloprid in tomatoes in previously published studies was 0.16–0.29 mg/kg [30]. Several methods for the detection of thiacloprid in environmental and food samples have been shown to be as quite reliable (verified by the standard HPLC method), such as the chemiluminescent enzyme immunoassay (ECL EIA), which was developed based on horseradish peroxidase [31]. Thiacloprid was determined along with other neonicotinoid pesticides in real samples, and methanol was also used as an extraction solvent, as in our study, which allows the complete removal of analytes from cartridges, and it was also found that the matrix effect depends on the type of sample [32]. Chlorantraniliprole is a second-generation anthranilic diamide insecticide that acts on insect ryanodine receptors and causes death due to the uncontrolled release of calcium ion stores in the muscle cells [33]. Methomyl residues after treating mint were determined using UPLC-MS, and a higher amount was found in the root (15.35–79.52 μg/kg) compared to the mixed leaves and stem (3.31–44.54 μg/kg) [34]. Quantification of methomyl in different samples was performed using HPLC, but with a one-step post-column derivatization reaction and a fluorescence detector [35]. In a recent study, methomyl was determined in a tomato fruit at levels ranging from 0.003 to 2.06 mg/kg [36]. Metalaxyl and its degradation products have been studied using lettuce and sunflower plants as examples in GC-MS and IR and using NMR [37]. Its quantification in some real samples (grapes) was performed using RP-HPLC UV-DAD, and the value found was 2 mg/kg [38]. GC-MS analysis was proved to be good for the determination of metalaxyl in strawberries [39]. HPLC-DAD was previously reported for the determination of metalaxyl in tomatoes [40]. The matrix effect in tomatoes was reduced in our study using QuEChERS [41].

As for the quantification of pesticide residues in real cucumber samples, the situation was worse than in the case of tomatoes. Among the three pesticides (cymoxanil, famoxadone, and thiamethoxam), only cymoxanil could be quantified in both samples (K1 and K2) (Figure S2). Thiamethoxam was detected in real samples spiked with pesticides, but its quantification was not possible because a standard curve could not be established. Famoxadone could not be detected in real samples spiked with pesticides, nor could a standard curve be established. The determined concentration of cymoxanil was higher in the K1 sample (1.25 mg/kg) than in the K2 sample (0.81 mg/kg).

Grapes are widely chemically treated against pests, and there are a variety of compounds for this purpose (abamectin, cymoxanil, metalaxyl-M, pyraclostrobin, propiconazole, and tebuconazole). Of these pesticides in the UV range of 180–400 nm, only cymoxanil was quantified in the spiked real samples of grapes examined (Figure S3). Unlike cucumber, cymoxanil was quantified in the real sample without the use of QuEChERS kits for highly pigmented substances (0.31 mg/kg). Cymoxanil was previously found in grape juices using QuEChERS and Liquid Chromatography-Tandem Mass Spectrometry [42].

Pepper as a plant is treated with several pesticides: acetamiprid, boscalid, emamectin benzoate, metalaxyl, pendimethalin, and pyraclostrobin, and some of them were detected and quantified in the real samples spiked with the pesticides (PP1 and PP2). Emamectin (without the benzoate), due to dissociation in the aqueous medium, was detected and quantified only in sample PP1 (13.53 mg/kg) (Figure S4). Emamectin benzoate was previously detected in rice and rice-growing areas by means of UPLC/MS/MS [43], and in fruits and vegetables by means of LC-MS/MS [44]. Acetamiprid was detected and quantified in both real samples: PP1 (0.025 mg/kg) and PP2 (0.008 mg/kg) (Figure S4). Previously, it was determined in vegetables using the Indirect Competitive Chemiluminescence Enzyme Immunoassay [45], in lettuce using a bioelectric cell biosensor-based system [46], and in sweet cherries (0.111–0.529 mg/kg) using the HPLC-UV DAD system [47]. A preliminary method has been developed for the rapid in situ determination of acetamiprid in fruits and vegetables using the surface enhanced Raman scattering (SERS) method based on the Au@AgNPs substrate [48].

The MRLs of selected pesticides in the investigated fruits and vegetables differ between the international standards. According to the EU standard, only the values found for pesticide residues of acetamiprid in peppers and metalaxyl in tomatoes are within the recommended values (0.3, and 0.2 mg/kg, respectively). The determined values of the remaining selected pesticides in the examined and not washed fruits and vegetables are outside the recommended values (cymoxanil in cucumbers 0.01 mg/kg, cymoxanil in grapes 0.01 mg/kg, emamectin-benzoate in peppers 0.02 mg/kg, chlorantraniliprole in tomatoes 0.6 mg/kg, methomyl in tomatoes 0.02 mg/kg, and thiacloprid in tomatoes 0.5 mg/kg).

### 2.2. Computational Studies (EIIP Calculation, Conformational Search, Molecular Docking, and ADMET) of Selected Pesticides and Acetylcholine Esterase from Mus musculus and Homo sapiens

The pesticides selected had AQVN values falling within the intervals of 2.614–3.167 (Table 2). The absolute values of EIIP range from 0.006 for methomyl to 0.100 for cymoxanil. Previous studies have shown that methomyl [49] and cymoxanil [50] exhibit AChE-inhibitory activity. Inhibitor specificity between the AChE of insects and mammals contributes to selective toxicity [51]. It is generally safer for insecticides to have a higher affinity for insect AChE than human AChE.

Before molecular docking, all selected pesticides were prepared in MacroModel. The global minimum of each pesticide was used for molecular docking. The global minimum energies and repeats of the studied structures are listed in Table 3.

After optimization, the selected pesticides were subjected to molecular docking against acetylcholinesterase from *Mus musculus* and *Homo sapiens*, the sequences of which have an identity of 89.80% using BLASTP (Protein BLAST: search protein databases using a protein query (nih.gov)), and the values of Glide scores from molecular docking are given in Table 3. Acetylcholine esterase was chosen because of its involvement in numerous cholinergic pathways in the central and peripheral nervous systems, the inactivation of which, induced by various inhibitors, leads to its accumulation, hyperstimulation of nicotinic and muscarinic receptors, and disrupted neurotransmission [21]. According to the performed molecular docking studies, the best binder for acetylcholinesterase from *Mus musculus* is emamectin benzoate as emamectin, and the worst binder is metalaxyl. In the case of *Homo sapiens* acetylcholinesterase, the best binder is acetamiprid, and the worst binder is methomyl.

Interestingly, the pesticides bind at the same site in *Mus musculus* and *Homo sapiens*. Acetamiprid has a common contact in *M. musculus* and *H. sapiens*: His 381. Leu 380 in *M. musculus* and Trp 385 and Gln 527 in *H. sapiens* are other contacts (Figure S5). Cymoxanil has two common contacts: Gln 527 and His 381, and additional contacts: Tyr 382 and Leu 380 in *M. musculus*, and Ala 528 in *H. sapiens* (Figure S6). Emamectin has two common contacts to both structures (Leu 386 and His 381), and additional contacts: Arg 525, Asp 400 in *H. sapiens*, and Gln 527, Tyr 382, and Arg 521 in *M. musculus* (Figure S7). Thiacloprid has two common contacts to both structures: Gln 527 and His 381, with the additional contact Phe 531 in *M. musculus* (Figure S8). Methomyl has two common contacts to both structures: His 381 and Leu 380, and additional contacts: Tyr 382, Arg 525 and Ala 528 in *H. sapiens*, and Phe 531 and Phe 535 in *M. musculus* (Figure S9). Metalaxyl has a common contact with both structures: Gln 527, and an additional contact in the case of *M. musculus*: His 381 (Figure S10). Chlorantraniliprole has three common contacts to both structures: Leu 380, His 381 and Gln 527, with one additional contact in the case of *H. sapiens*: Ala 528 (Figure S11).

### 2.3. Molecular Docking Studies of Approved Alzheimer’s Medicines and Selected Pesticides against Acetylcholine Esterase from H. sapiens

Drugs used to treat Alzheimer’s disease (donepezil, rivastigmine and galantamine) that target AChE [52] fall within the AQVN range of 2.5 and 2.667. Analyzed pesticides and drugs for Alzheimer’s disease shared a common target—acetylcholine esterase. The crystal structure of donepezil with acetylcholine esterase from *H. sapiens* was used for our docking studies, bearing in mind its binding site from chain B including interactions with Ser 293, Trp 286 and Trp 86 [53]. Donepezil redocking provided a Glide score of −3.90 kcal/mol. Two other approved medicines for the treatment of Alzheimer’s disease (rivastigmine and galantamine) provided better results for galantamine (−5.38 kcal/mol) and worse results for rivastigmine (−2.94 kcal/mol). Regarding the selected pesticides, the obtained values (in kcal/mol) were −4.84 (acetamiprid), −3.68 (cymoxanil), −4.63 (chlorantraniliprole), −4.83 (metalaxyl), −3.55 (methomyl), and −4.19 (thiacloprid). Emamectin was not possible to dock into the chosen binding site. Therefore, all selected pesticides (except emamectin) are better inhibitors of acetylcholine esterase than rivastigmine. Based on the values for acute oral LD_50_ obtained for rats (mg/kg) of selected pesticides, only chlorantraniliprole can be considered (>5000) [54] for further studies, and maybe a less toxic modification can be designed in the future. Other investigated pesticides with very low acute LD_50_ values show high toxicity to mammals [54].

### 2.4. In Silico ADMET Studies of Selected Pesticides

The toxicity of the compounds was assessed using Lipinski’s Rule of Five [55], which includes the molecular weight (<500 Da), number of hydrogen-bond acceptors (≤10) and donors (≤5), the octanol/water partition coefficient (≤5), and Jorgensen’s rule of three [56], which includes logS (>−5.7), PCaco (>22 nm/s), and primary metabolites (PM) (<7). The violations of these rules are essential for the optimization of biologically active compounds and should not exceed 1.

Table 4 shows the ADMET properties of the selected compounds (methomyl, thiacloprid, metalaxyl, chlorantraniliprole, acetamiprid, emamectin benzoate, cymoxanil) predicted by QikProp and includes the following parameters: molecular weight (MW), number of rotatable bonds (RB), dipole moment (DM), molecular volume (MV), number of hydrogen donors (DHB), number of hydrogen acceptors (AHB), polar surface area (PSA), octanol/water partition coefficient (log P), water solubility (log S), apparent Caco-2 cell permeability (PCaco), number of probable primary metabolic reactions (PM), percentage of oral absorption by humans (%HOA), and violations of the rules of three (VRT) and rule of five (VRF). The theoretical calculations of the ADME parameters are presented in Table 4, along with the Lipinski and Jorgensen rule violations. According to QikProp, the calculation for emamectin benzoate is not possible because this compound consists of two molecules. Thus, it can be assumed that according to the predictions of the ADMET properties, all compounds are orally active.

The toxicity profiles of selected pesticide compounds were predicted using SwissADME. The results are presented in Table 5. The web-based SwissADME tool has access to several models for the prediction of physicochemical parameters (since ADME is directly related to the properties of the molecule, and individual pharmacokinetic behavior) and medicinal chemistry. The SwissADME descriptors for toxicity are TPSA—polar surface area; Consensus logP_o/w_—consensus prediction of lipophilicity; log S_-ali_—predicted water solubility; P_-gp_—permeability glycoprotein-1; Lipinski—Lipinski rule of five (fail not more than 1 criterion): MW < 500 g/mol, CLOGP < 5 (MLOGP < 4.15), number of H-bond donors ≤ 5, number H-bond acceptors ≤ 10; Egan—Egan Rule ALOGP98 < 6 and TPSA < 132 A^2^ [57]; PAINS—promiscuous fragments [58]; and SA_score_—synthetic accessibility score [59].

## 3. Materials and Methods

### 3.1. Chemicals and Reagents

Certified standards of pesticides (chlorantraniliprole, methomyl, metalaxyl, thiacloprid, acetamiprid, emamectin benzoate, cymoxanil, thiamethoxam, propiconazole, abamectin, boscalid, famoxadone, metalaxyl-M, pendimethalin, pyraclostrobin, tebuconazole) were purchased from Dr Ehrenstorfer (Augsburg, Germany). Methanol and glacial acetic acid of HPLC grade were purchased from J. T. Baker (Landsmeer, The Netherlands). Anhydrous magnesium sulfate was purchased from VWR BDH CHEMICALS (Suwanee, GA, USA), and anhydrous sodium acetate was purchased from Zdravlje (Leskovac, Republic of Serbia). PSA was purchased from UCT, Inc. (Bristol, PA, USA). QuEChERS kits for the removal of highly pigmented substances (roQ^TM^ QuEChERS dSPE Kit-15 mL CT) were purchased from Phenomenex, Inc. (Torrance, CA, USA).

Stock solutions of pesticides: chlorantraniliprole (1.0597 mg/mL), methomyl (1.2969 mg/mL), metalaxyl (1.2848 mg/mL), thiacloprid (0.22572 mg/mL), acetamiprid (1.2553 mg/mL), emamectin benzoate (1.0303 mg/mL), cymoxanil (1.1952 mg/mL), thiamethoxam (0.23928 mg/mL), propiconazole (0.25318 mg/mL), abamectin (0.2652 mg/mL), boscalid (0.23366 mg/mL), famoxadone (0.24058 mg/mL), metalaxyl m (1.1026 mg/mL), pendimethalin (0.30215 mg/mL), pyraclostrobin (1.0673 mg/mL), and tebuconazole (0.23668 mg/mL) were prepared in methanol and stored at 4 °C. Standard working solutions were prepared daily by means of the appropriate dilution of each stock solution with methanol. The standard working solution of each pesticide was used as a spiking solution and to prepare the calibration standard solutions.

### 3.2. Preparation of Real Samples of Agricultural Products (Tomatoes, Cucumbers, Peppers, and Grapes)

The real samples of agricultural products (tomatoes, cucumbers, peppers, and grapes) were prepared by means of two methods. The only difference between the second and the first method is the use of QuEChERS kits for highly pigmented substances in the second method. The methods are a variation of the previously published method [60].

#### 3.2.1. Method 1

Agricultural products (tomatoes, cucumbers, peppers, and grapes) were taken from an individual farm in the Republic of Serbia, treated with pesticide formulations, and crushed with an electric blender. Then, 15.1183 g, 15.0570 g, 15.1109 g, and 15.2371 g of the sample (tomatoes, cucumbers, peppers, and grapes, respectively) were placed in a centrifuge tube (50 mL) and 15 mL of methanol with 1% *v/v* glacial acetic acid, 6 g of anhydrous magnesium sulfate, and 1.5 g of anhydrous sodium acetate were added. The tube was then placed in an ice ultrasonic bath and mixed for 21 min. Centrifugation was then performed for 5 min (Hermle L 306, rcf = 4430). The solid was separated from the supernatant and then acidified methanol was added again to the solid residue and centrifuged for 5 min. Then, 12 g of anhydrous magnesium sulfate and 4 g of PSA were added to the resulting mixed extracts, cooled, and centrifuged for 10 min. In all cases, except tomatoes, 6 g of anhydrous magnesium sulfate, and acidified methanol were then added and centrifuged. The procedure was repeated three times with 10 g of anhydrous magnesium sulfate and one more time with 6 g of anhydrous magnesium sulfate. The supernatant was filtered through a microfilter (45 μm) into a volumetric flask (25 mL for tomatoes and cucumbers and 10 mL for peppers and grapes). The volumetric flasks were stored in the refrigerator.

#### 3.2.2. Method 2

Agricultural products (tomatoes, cucumbers, peppers, and grapes) were taken from an individual farm in the Republic of Serbia, treated with pesticide formulations, and crushed with an electric blender. Then, 15.0876 g, 15.0626 g, 15.0082 g, and 15.1402 g of the sample (tomatoes, cucumbers, peppers, and grapes, respectively) were placed in a centrifuge tube (50 mL) and 15 mL of methanol containing 1% *v/v* glacial acetic acid was added, followed by 6 g of anhydrous magnesium sulfate and 1.5 g of anhydrous sodium acetate. The tube was then placed in an ice ultrasonic bath and mixed for 21 min. Centrifugation was then performed for 5 min (Hermle L 306, rcf = 4430). The solid was separated from the supernatant and then acidified methanol was added again to the solid residue and centrifuged for 5 min. Then, 12 g of anhydrous magnesium sulfate and 4 g of PSA were added to the obtained mixed extracts, cooled, and centrifuged for 10 min. The solid was separated from the supernatant, and then 15 mL of acidified methanol was added and centrifuged for 10 min. The liquid supernatants were mixed and centrifuged for another 10 min. To the supernatant obtained, a QuEChERS kit for highly pigmented samples containing 900 mg of MgSO_4_, 150 mg of PSA, and 45 mg of GCB was added and centrifuged for 10 min. The supernatant was filtered through a microfilter (45 μm) into a volumetric flask (25 mL). The volumetric flasks were stored in the refrigerator.

### 3.3. Preparation of Spiked Samples

Three different concentrations of pesticides were used for spiking the real samples. For tomatoes, 2.5 mL of the solution of the prepared real sample and 1 mL of chlorantraniliprole standard solution, 1 mL of methomyl standard solution, 1 mL of metalaxyl standard solution and 1 mL of thiacloprid standard solution were added to a volumetric flask (10 mL) for both prepared samples, and methanol was added up to the mark. For cucumber, for both prepared samples, 2.5 mL of the prepared real sample solution and 1 mL of cymoxanil standard solution, 1 mL of famoxadone standard solution, 1 mL of thiamethoxam standard solution and methanol up to the mark were added to a volumetric flask (10 mL). For peppers, 2.5 mL of the solution of the prepared real sample according to method 2 (1 mL of the real sample according to method 1), 1 mL of acetamiprid standard solution, 1 mL of boscalid standard solution, 1 mL of emamectin benzoate standard solution, 1 mL of metalaxyl standard solution, 1 mL of pendimethalin standard solution, 1 mL of pyraclostrobin standard solution and methanol were added to a volumetric flask (10 mL) up to the mark. For grapes, 2.5 mL of the solution of the prepared real sample according to method 1 (1 mL of the real sample according to method 1) and 1 mL of abamectin standard solution, 1 mL of cymoxanil standard solution, 1 mL of metalaxyl-M standard solution, 1 mL of pyraclostrobin standard solution, 1 mL of propiconazole standard solution and 1 mL of tebuconazole standard solution and methanol were added to a volumetric flask (10 mL) up to the mark.

### 3.4. HPLC-DAD Analysis

An Agilent Technologies 1100 series chromatograph equipped with a degasser, quaternary pump, thermostated column (C18, 4.6 × 150 mm, 5 μm), and a UV/VIS detector was used to quantify pesticide residues. A gradient profile with two solvents at 25 °C was used. The solvents used were as follows: solvent A: water and solvent B: methanol. A flow rate of 1.0 mL/min was applied and 20 μL of the sample was injected. The gradient was as follows: 0 min—50% B; 45 min—10% B; 60.1–50% B [61]. The wavelength of the diode array detector was set at 245 nm for pesticide residue monitoring. Pesticides were identified by comparing their UV spectra and retention times with those of the standards. The concentrations of all of the pesticide residues in the extract were quantified using the standard curves and expressed as mg per kg real sample (mg/kg).

### 3.5. Electron-Ion Interaction Potential (EIIP)/Average Quasi-Valence Number (AQVN)

Specific recognition and targeting between interacting biological molecules at distances > 5 Å are determined by the AQVN (average quasi-valence number) and the EIIP (electron–ion interaction potential) derived from the general model pseudopotential [62]:

EIIP = 0.25(Z*/(2π))Sin(1.04πZ*)

where Z* is the AQVN determined by:

$$Z^{*}=\frac{1}{N}\overset{m}{\underset{i=1}{\sum }}n_{i}Z_{i}$$

where *Z_i_* is the valence number of the *i*th atomic component, *n_i_* is the number of atoms of the *i*th component, *m* is the number of atomic components in the molecule, and *N* is the total number of atoms. The Z* and EIIP values are expressed in Rydberg units (Ry).

AQVN and EIIP are unique physical properties that characterize long-range interactions between biological molecules among the molecular descriptors [63]. It has been shown that the EIIP and AQVN of organic molecules strongly correlate with their biological activity (mutagenicity, carcinogenicity, toxicity, antibiotic, and cytostatic activity, etc.) [64,65].

### 3.6. Unconstrained Conformational Search

**Figure 1:**
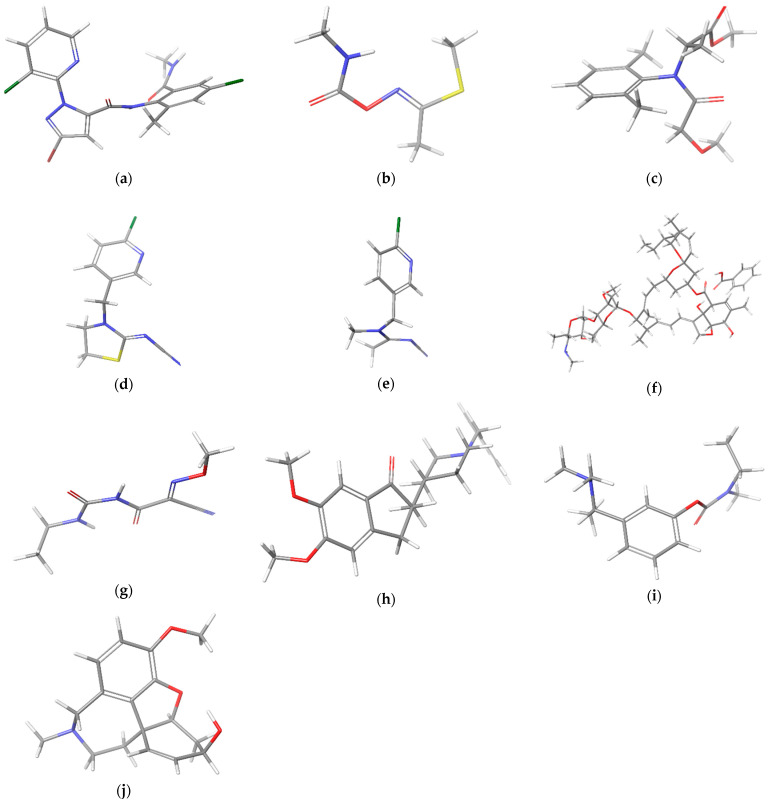
Structures of determined pesticides by HPLC-DAD and computationally investigated pesticides (**a**–**g**) and approved medicines against Alzheimer’s disease (**h**–**j**): (**a**) chlorantraniliprole, (**b**) methomyl, (**c**) metalaxyl, (**d**) thiacloprid, (**e**) acetamiprid, (**f**) emamectin benzoate, (**g**) cymoxanil, (**h**) donepezil, (**i**) rivastigmine, (**j**) galantamine.

Conformational analysis of selected pesticides (chlorantraniliprole, methomyl, metalaxyl, thiacloprid, acetamiprid, emamectin benzoate, and cymoxanil) (Figure 1) was performed using MacroModel under Schrodinger Suite 2021-1 and Maestro v. 12.7.156 as the interface. Donepezil, rivastigmine and galantamine (Figure 1) conformational analysis was performed using Schrodinger Suite 2022-3 and Maestro v. 13.3 as the interface. Chloroform was used as the solvent. The minimizations were initially performed with charges from the force field (MMFFs), the cut-off was extended, the minimization method was TNCG (Truncated Newton Conjugate Gradient), and the number of maximum iterations was set to 10000, with gradient convergence, and a threshold of 0.05. The torsion sampling for the conformational search was MCMM (Monte Carlo Multiple Minimum) with automatic setting during the calculation, and torsion sampling options were intermediate. The maximum number of steps was 10,000, with 100 steps per rotatable bond. The number of structures to be stored for each search was 100, the energy window for storing structures was 21 kJ/mol, and the maximum atom deviation cut-off was 0.5 Å.

### 3.7. Molecular Docking Studies

Molecular docking studies with selected pesticides (chlorantraniliprole, methomyl, metalaxyl, thiacloprid, acetamiprid, emamectin, and cymoxanil) were performed using acetylcholinesterase as a target from *Mus musculus* and from *Homo sapiens*. In addition, molecular docking was performed with approved Alzheimer’s medicines (donepezil, rivastigmine, galantamine) and selected pesticides (chlorantraniliprole, methomyl, metalaxyl, thiacloprid, acetamiprid, emamectin, and cymoxanil) into the binding site of donepezil in acetylcholine esterase from *H. sapiens* [53]. In all cases, the crystal structures were obtained from the Protein Data Bank (entry ID for *Mus musculus* 5DTI [66], and *Homo sapiens* 4EY7 [53]). Acetylcholinesterase alone was prepared for docking using the Protein Preparation and Refinement tool of Schrodinger Suite 2022-3. Previously optimized structures of selected pesticides and approved medicines in MacroModel were ligands in the molecular docking studies. Molecular docking was performed using Glide under Schrodinger Suite 2022-3.

### 3.8. ADMET In Silico Studies

ADMET parameters of the compounds (methomyl, thiacloprid, metalaxyl, chlorantraniliprole, acetamiprid, emamectin benzoate, cymoxanil) were calculated using QikProp v7.0 software running in normal mode (Schrödinger, Inc., New York, NY, USA). The web-based tool SwissADME [67] was also used to predict toxicity.

### 3.9. Determination of log D and pKas Values of Experimentally Determined Pesticides

ACD/Labs PhysChem Suite v14 (Advanced Chemistry Development Inc. Toronto, Canada) was used for the fast and accurate determination of pKa values and distribution coefficients (log D) for experimentally determined pesticides (methomyl, thiacloprid, metalaxyl, chlorantraniliprole, acetamiprid, emamectin benzoate, cymoxanil). The determination of pKas values was based on Hammett’s equation, and log D values were calculated on different pH values [68]. These parameters were used for the optimal conditions for the simultaneous extraction of the selected pesticides from the agricultural products.

## 4. Conclusions

Selected pesticides (chlorantraniliprole, methomyl, metalaxyl, thiacloprid, acetamiprid, emamectin benzoate, and cymoxanil) were determined in agricultural products (tomatoes, cucumbers, peppers and grapes) using QuEChERS methodology and HPLC-DAD. Thiacloprid was found in the real sample of tomatoes without spiking. The HPLC-DAD method is fast because the pesticides can be determined in less than 15 min, and reliable because of the good linearity of standard curves. According to the EU standard, only the values found for pesticide residues of acetamiprid in peppers and metalaxyl in tomatoes are within the recommended values (0.3, and 0.2 mg/kg, respectively). AQVN/EIIP was shown as a good prediction tool for the behaviour of selected pesticides against acetylcholine esterase. These selected pesticides against acetylcholine esterase in silico show the same region for binding both in *Mus musculus* and *Homo sapiens* with some differences. Based on the values for acute oral LD_50_ obtained for rats (mg/kg) regarding pesticides, only chlorantraniliprole stands out as a potential candidate (>5000) for further studies regarding the treatment of Alzheimer’s disease, and less toxic modifications could potentially be designed in the future.

## Figures and Tables

**Table 1:** Linear regression equations, correlation coefficients, and retention times of selected pesticides.

Pesticides	Linear Regression Equations	Correlation Coefficients (R^2^)	Retention Time (min)
**Chlorantraniliprole**	y = 30.083x − 54.359	0.9826	14.25
**Methomyl**	y = 21.122x − 32.026	0.9986	1.8
**Metalaxyl**	y = 2.2131x + 14.37	0.9969	9.9
**Thiacloprid**	y = 321.89x + 167.29	0.9984	2.844
**Acetamiprid**	y = 102.59x − 107.96	0.9977	2.427
**Emamectin benzoate**	y = 1.165x + 5.8913	0.9785	1.17
Cymoxanil *	y = 24.121x + 4.4977 y = 28.514x − 21.374	0.9978 0.9996	2.846 2.800

* The first calibration curve is related to the series of standard solutions used also for spiking real samples of cucumbers. The second calibration curve is related to the series of standard solutions also used for spiking real samples of grapes.

**Table 2:** Investigated pesticides, their classes, molecular formulae, Z*, absolute EIIP, log D and pKa values.

Pesticide	Class	Molecular Formula	Z* (Ry)	EIIP’ (Ry)	log D	pKa_1_	pKa_2_	pKa_3_
**Acetamiprid**	Neonicotinoid insecticide; pyridylmethylamine neonicotinoid insecticide	C_10_H_11_ClN_4_	2.769	0.041	1.55	−2.47	−0.44	/
**Chlorantraniliprole**	Diamide insecticide; pyridylpyrazole insecticide	C_18_H_14_BrCl_2_N_5_O_2_	3.000	0.044	3.64	10.19	14.77	/
**Cymoxanil**	Cyanoacetamide oxime fungicide; urea fungicide; nitrile fungicide	C_7_H_10_N_4_O_3_	3.167	0.100	0.67 (pH = 2), −0.21 (pH = 5.5), −0.96 (pH = 6.5), −1.26 (pH = 7.4), −1.33 (pH = 10)	−1.54	5.59	16.14
**Emamectin Benzoate**	Avermectin class insecticide	C_56_H_81_NO_15_	2.614	0.080	3.38 (pH = 2), 3.63 (pH = 5.5), 5.16 (pH = 7.4), 6.46 (pH = 10)	8.71	12.42	13.37
**Metalaxyl**	Anilide fungicide; acylamino acid fungicide	C_15_H_21_NO_4_	2.683	0.065	1.76	1.41	/	/
**Methomyl**	Carbamate insecticide; oxime carbamate insecticide; carbamate acariicide; oxime carbamate acaricide	C_5_H_10_N_2_O_2_S	2.90	0.006	0.6	−1.25	13.27	/
**Thiacloprid**	Neonicotinoid insecticide; pyridylmethylamine neonicotinoid insecticide; thiazolidine insecticide	C_10_H_9_ClN_4_S	3.04	0.059	2.2	0.01	/	/

**Table 3:** Global minima energies and repeats of the investigated pesticides, and Glide scores of selected pesticides against acetylcholine esterase from *Mus musculus* and *Homo sapiens*.

Pesticide	Global Minimum Energy (kJ/mol)	Number of Repeats	Glide Score (kcal/mol)	
			*Mus musculus*	*Homo sapiens*
**Acetamiprid**	−79.3	53	−4.69	−5.21
**Chlorantraniliprole**	91.1	15	−5.17	−4.94
**Cymoxanil**	−236.8	28	−3.9	−3.85
**Emamectin benzoate**	98.3	13	−5.76	−3.97
**Metalaxyl**	318.7	9	−1.37	−5.01
**Methomyl**	−82.4	65	−4.94	−2.82
**Thiacloprid**	−204	70	−4.55	−4.56

**Table 4:** Calculated absorption, distribution, metabolism, elimination, and toxicity (ADMET) parameters of the compounds.

Compound	MW	RB	DM	MV	DHB	AHB	PSA	logP	logS	PCaco	PM	%HOA	VRF	VRT	herg K+
**methomyl**	162.2	3	6.5	599.3	1	4	68.1	0.9	−1.8	1305	0	88	0	0	−3.4
**thiacloprid**	252.7	4	8.8	776.9	0	4	51.9	2.3	−2.9	1157	2	95	0	0	−4.3
**metalaxyl**	279.3	5	7.1	488.9	0	6.7	55.4	1.6	−1.2	3809	6	100	0	0	−3.5
**chlorantraniliprole**	483.1	3	5.9	1182.8	1	6	79.2	4.7	−6.7	1816	2	100	0	1	−5.9
**acetamiprid**	222.7	4	7.1	737.8	0	4	59.4	1.8	−2.2	927	2	91	0	0	−3.9
**emamectin benzoate**	1008.2	-	-	-	-	-	-	-	-	-	-	-	-	-	-
**cymoxanil**	198.2	5	12.8	713.5	1	6.7	121.4	14.1	−1.9	73	0	55	0	0	−3.1

**MW**: molecular weight; **RB**: number of rotatable bonds; **DM**: computed dipole moment; **MV**: total solvent-accessible volume; **DHB**: estimated number of hydrogen-bond donors; **AHB**: estimated number of hydrogen-bond acceptors; **PSA**: van der Waals surface area of polar nitrogen and oxygen atoms and carbonyl carbon atoms; **logP**: predicted octanol/water partition coefficient; **log S**: predicted aqueous solubility; **PCaco**: predicted apparent Caco-2 cell permeability; **PM**: number of likely metabolic reactions; **%HOA**: predicted human oral absorption percentage; **VRF**: number of violations of Lipinski rule of five (the rules are as follows: MW < 500, log P < 5, DHB ≤ 5, AHB ≤ 10, positive PSA value); **VRT**: number of violations of Jorgensen rule of three (the rules are as follows: log S > −5.7, PCaco > 22 nm/s, PM < 7); **herg K+**: Human Enter-a-go-go Related Gene (concern below −5).

**Table 5:** Calculated toxicity parameters of the selected pesticides using SwissADME.

Compound	MW	TPSA	C logP_o/w_	log S_-ali_	P_-gp_	Lipinski	Egan	PAINS	SA_score_
**methomyl**	162.2	75.9	0.8	−1.8	No	0	0	0	2.8
**thiacloprid**	252.7	77.6	1.7	−3.1	No	0	0	0	2.9
**metalaxyl**	279.3	55.8	2.0	−2.4	No	0	0	0	2.6
**chlorantraniliprole**	483.1	88.9	3.76	−6.4	No	0	0	0	3.1
**acetamiprid**	222.7	52.3	1.64	−2.1	No	0	0	0	2.4
**emamectin benzoate**	1008.2	199.2	4.6	−7.1	Yes	2	2	0	10
**cymoxanil**	198.2	103.6	−0.45	−2.3	No	0	0	0	2.8

**MW**: molecular weight; **TPSA**: polar surface area; **Consensus logP_o/w_**: consensus prediction of lipophilicity; **log S_-ali_**: predicted water solubility; **P_-gp_**: permeability glycoprotein-1; **Lipinski**: Lipinski rule of five (fail not more than 1 criterion): MW < 500 g/mol, CLOGP < 5 (MLOGP < 4.15), number of H-bond donors ≤ 5, number H-bond acceptors ≤ 10; **Egan**: Egan Rule ALOGP98 < 6 and TPSA < 132 A^2^; **PAINS**: promiscuous fragments; **SA_score_:** synthetic accessibility score.

## Data Availability

Data are contained within the article and Supplementary Material.

## Supplementary Materials

The following supporting information can be downloaded at: https://www.mdpi.com/article/10.3390/ijms24098003/s1.
