# Supplementary material for: In Silico Investigation of Selected Pesticides and Their Determination in Agricultural Products Using QuEChERS Methodology and HPLC-DAD
Source: Int J Mol Sci. 2023 Apr 28;24(9):8003. doi: 10.3390/ijms24098003 (PMC10179243; doi:10.3390/ijms24098003)
Supplement: Supplementary file 1 [file ijms-24-08003-s001.zip › ijms-2340847-supplementary.pdf]

Supplementary Material

In silico investigation of selected pesticides and their determination in agricultural products using QuEChERS methodology and HPLC-DAD

Stefan Petrović[1], Biljana Arsić[1,*], Ivana Zlatanović[1], Jelena Milićević[2,*], Sanja Glišić[2], Milan Mitić[1], Rada Đurović-Pejčev[3], Gordana Stojanović[1]

[1] Department of Chemistry, Faculty of Sciences and Mathematics, University of Niš, Višegradska 33, 18106 Niš, Republic of Serbia

[2] Laboratory for Bioinformatics and Computational Chemistry, Vinča Institute of Nuclear Sciences, Mike Petrovića Alasa 12-14, 11351 Vinča, Belgrade, Republic of Serbia

[3] Institute of Pesticides and Environmental Protection, Banatska 31b, 11080 Zemun, Belgrade, Republic of Serbia

*corresponding authors.

E-mails: Biljana.arsic@pmf.edu.rs (Biljana Arsić); jdjordjevic@vinca.rs (Jelena Milićević)

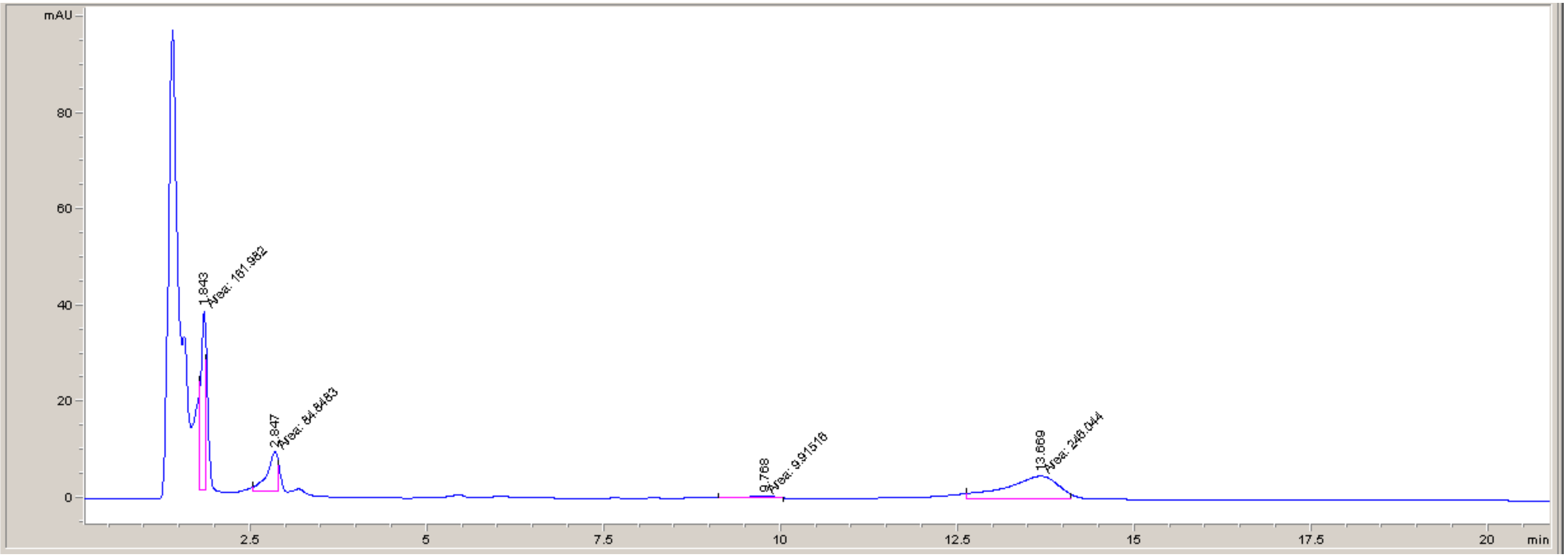
mAU
80
60
40
20
0
1.843
Area: 161.982
2.847
Area: 84.8483
9.768
Area: 9.91518
13.669
Area: 248.044
2.5
5
7.5
10
12.5
15
17.5
20
min

a)

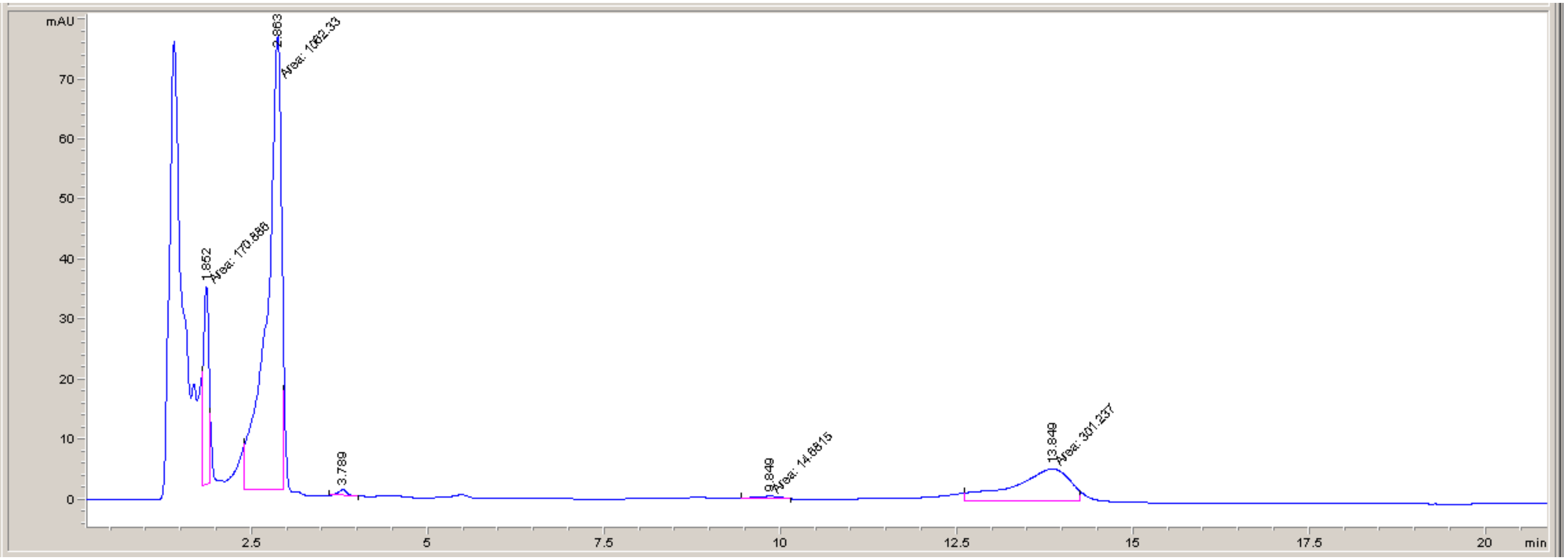

b)

Figure S1. Representative chromatograms of a real sample of tomatoes spiked with standard solutions of selected pesticides (chlorantraniliprole, methomyl, metalaxyl and thiacloprid) and prepared using a) Method 1, and b) Method 2.

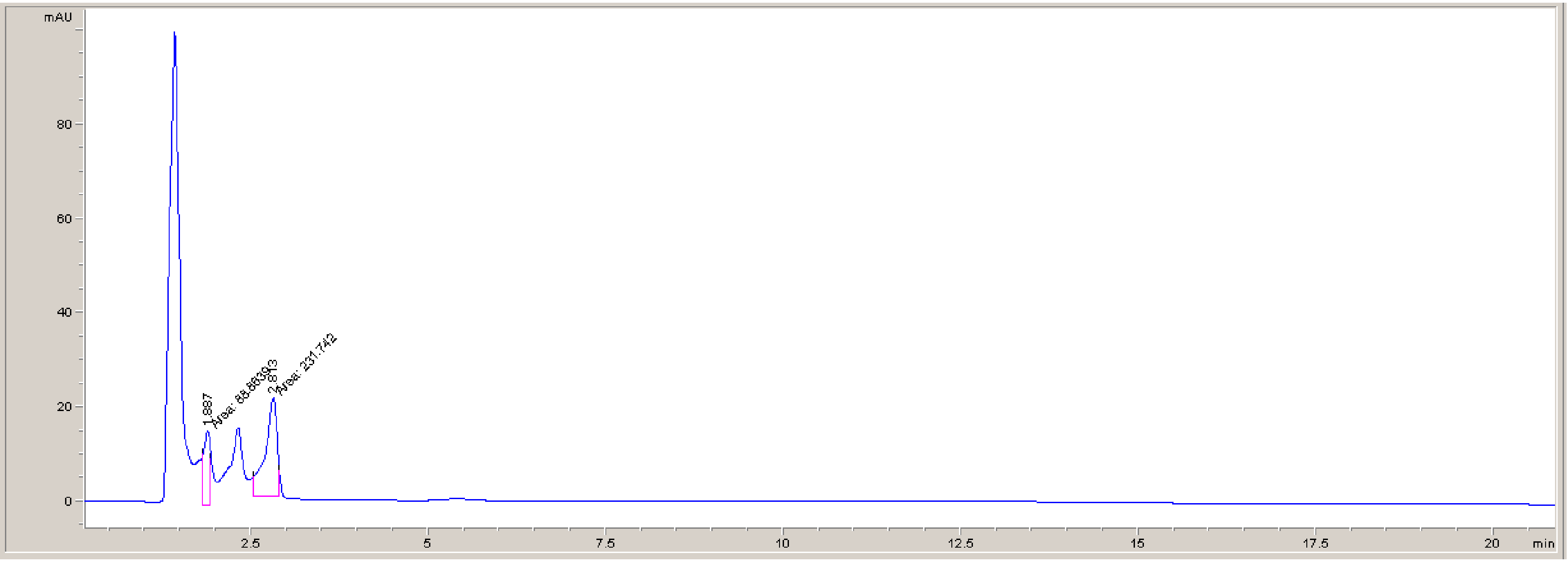

a)

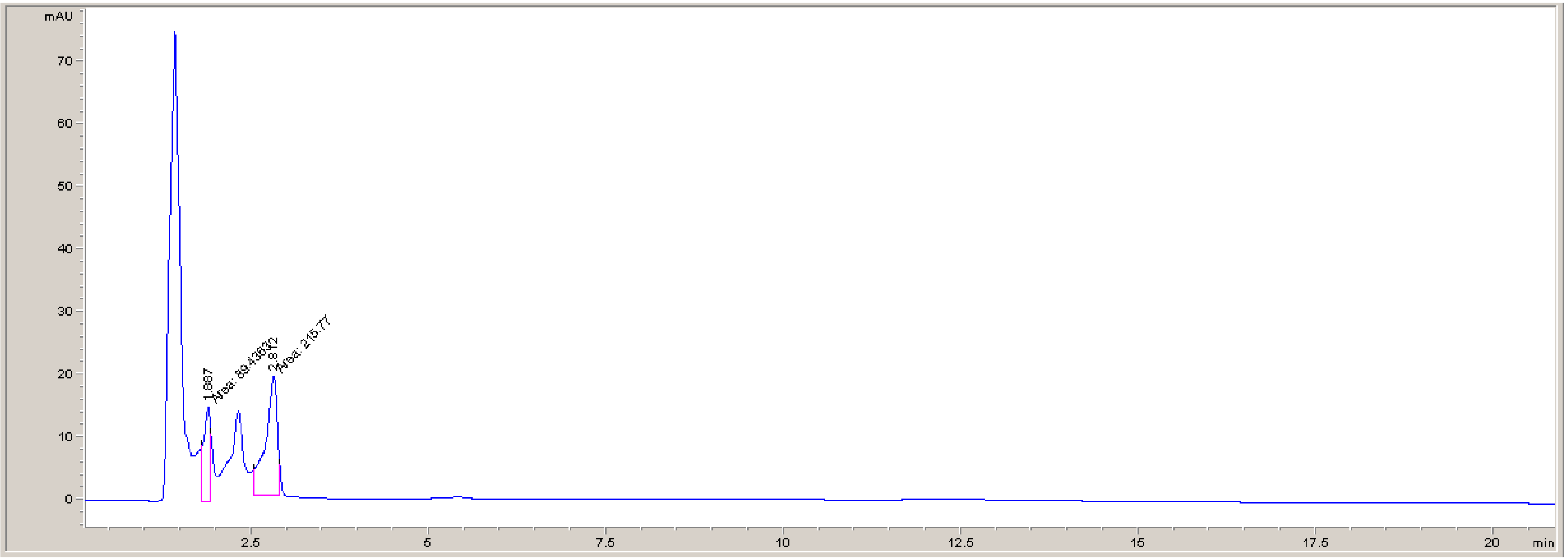

b)

Figure S2. Representative chromatograms of real sample of cucumber spiked with standard solutions of selected pesticides (cymoxanil, famoxadone, and thiamethoxam) and prepared using a) Method 1, and b) Method 2.

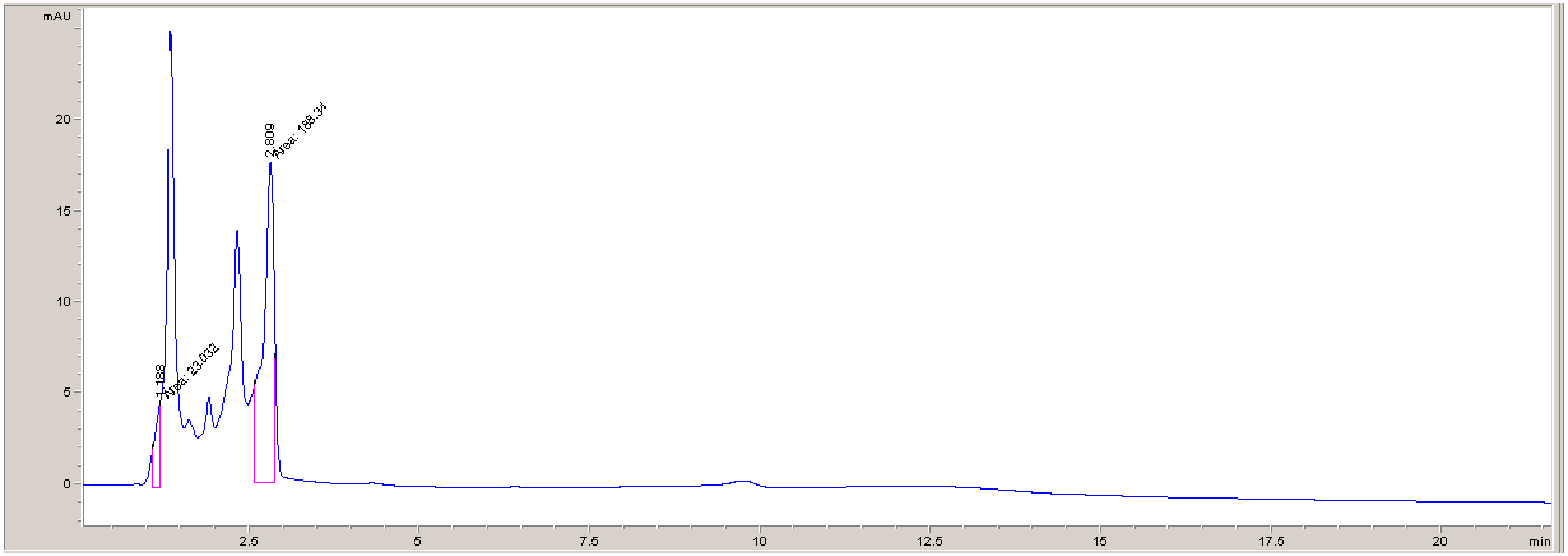
mAU
20
15
10
5
0
1.188
Area: 23.032
2.809
Area: 188.34
2.5
5
7.5
10
12.5
15
17.5
20
min

a)

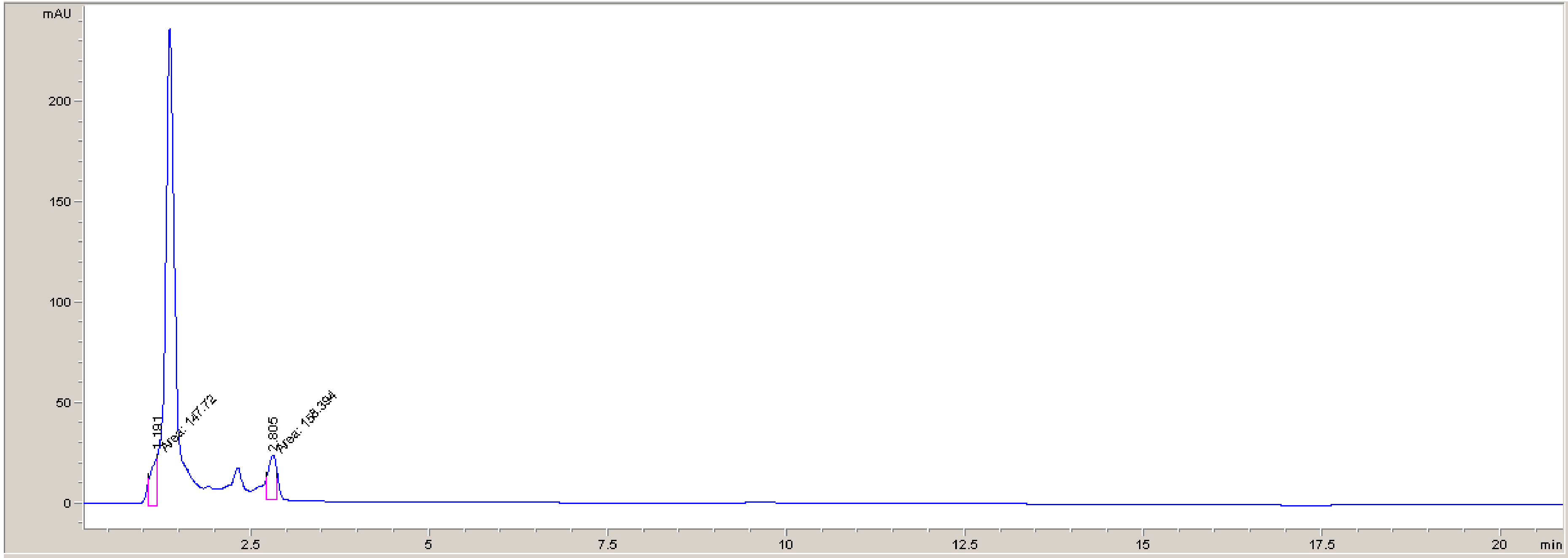

b)

Figure S3. Representative chromatograms of real sample of grapes spiked with standard solutions of selected pesticides (abamectin, cymoxanil, metalaxyl-M, pyraclostrobin, propiconazole, and tebuconazole) and prepared using a) Method 1, and b) Method 2.

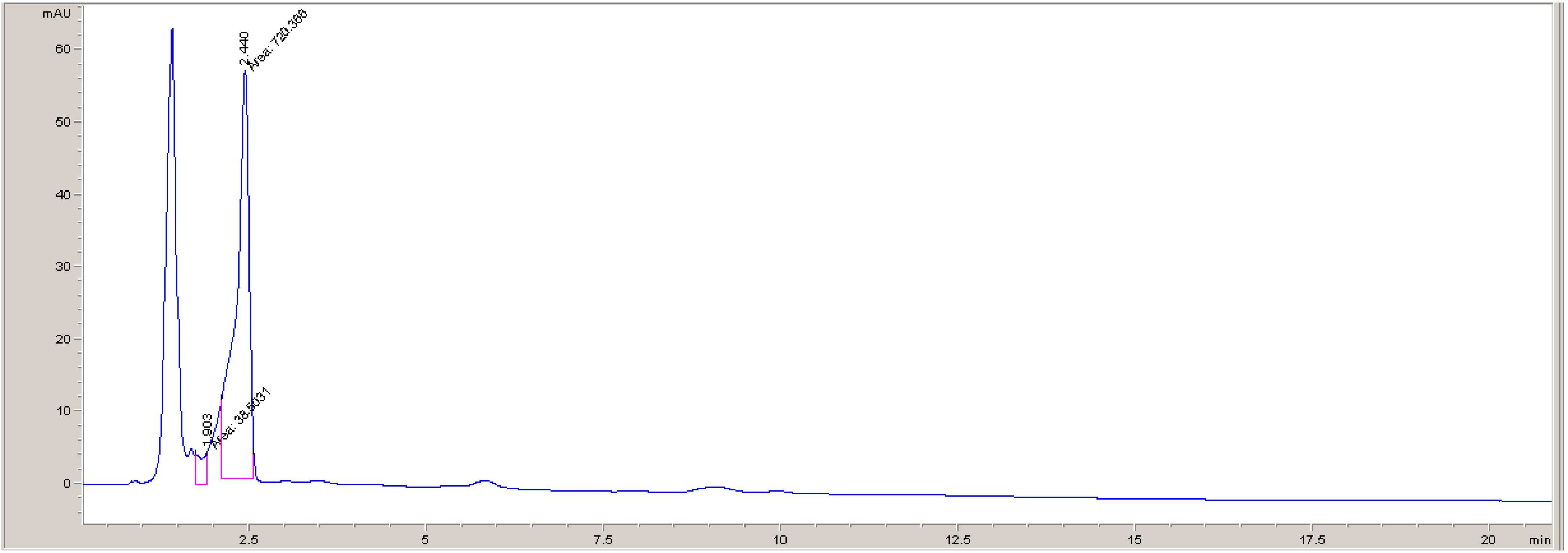

mAU
60
50
40
30
20
10
0
2.5
5
7.5
10
12.5
15
17.5
20
min
2.440
Area: 720.366
1.903
Area: 38.5031

a)

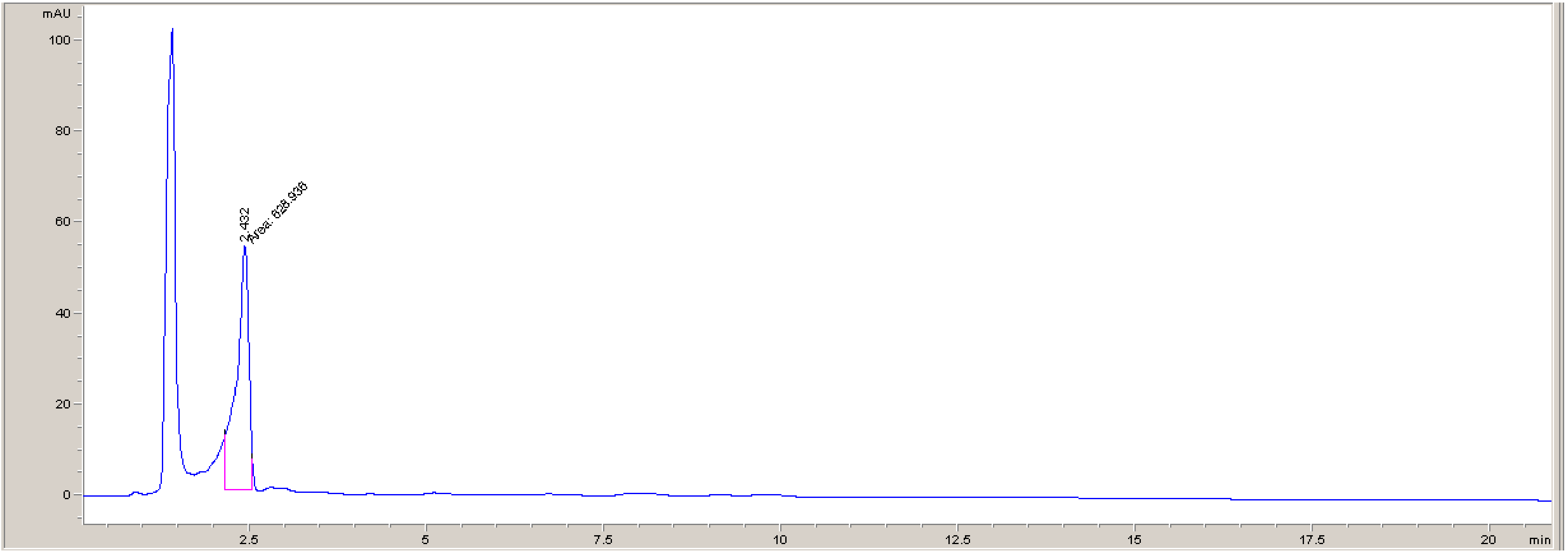

b)

Figure S4. Representative chromatograms of real sample of pepper spiked with standard solutions of selected pesticides (acetamiprid, boscalid, emamectin benzoate, metalaxyl, pendimethalin, and pyraclostrobin) and prepared using a) Method 1, and b) Method 2.

a)

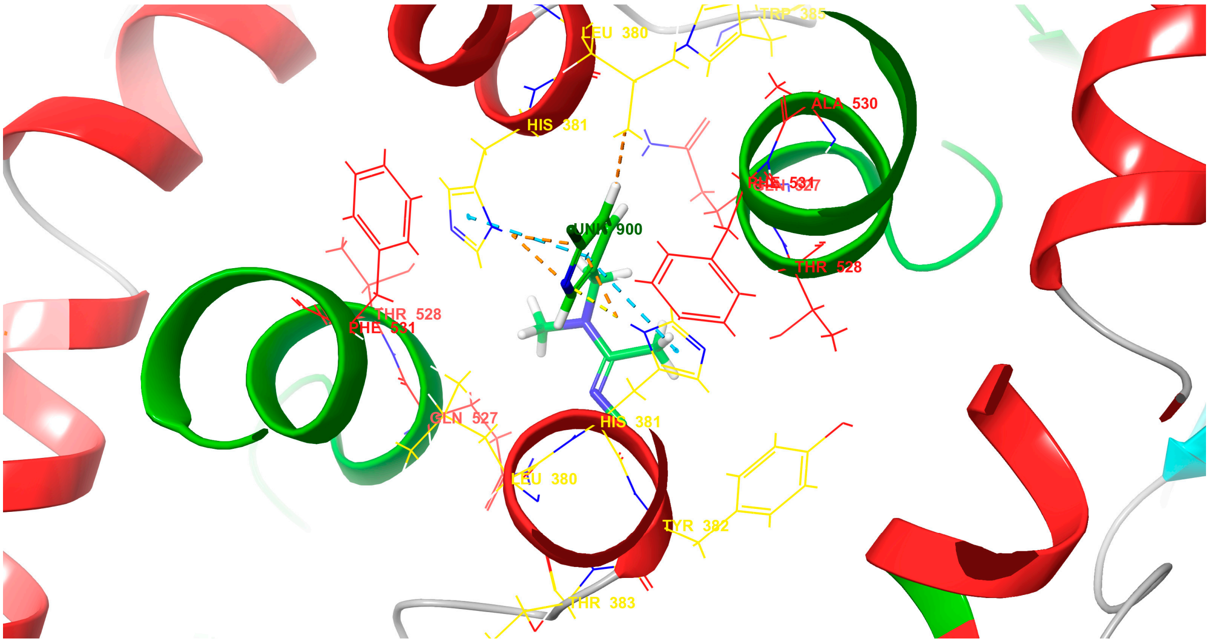

b)

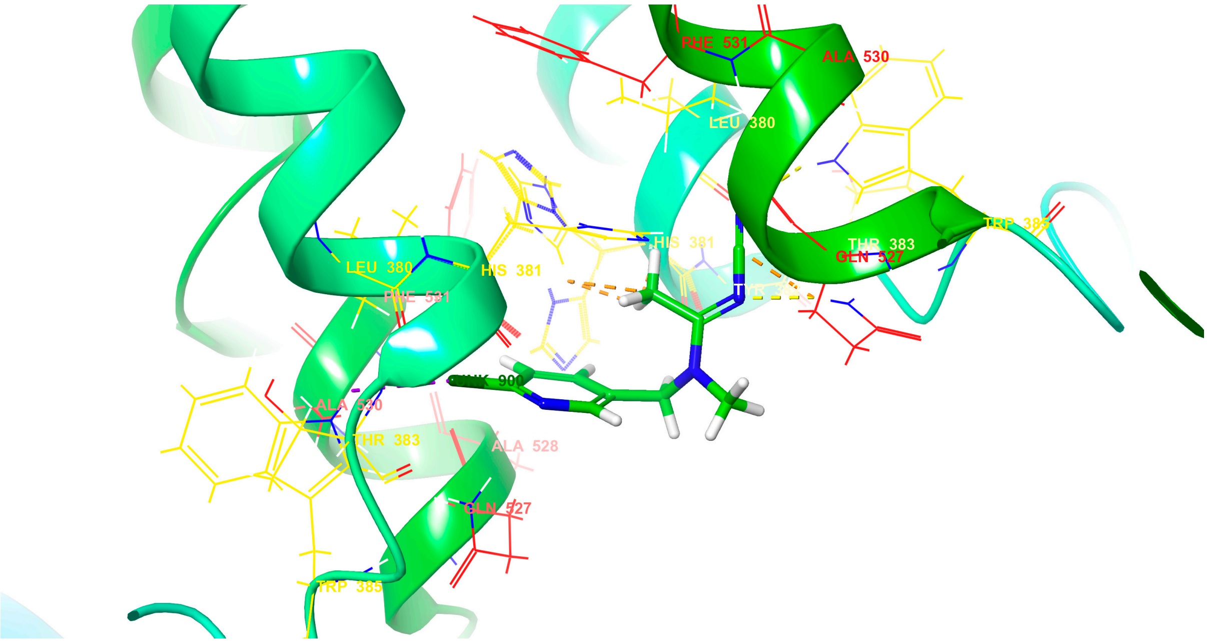

Figure S5. Graphic representations of docking acetamiprid into the structure of acetylcholine esterase from a) *Mus musculus*; b) *Homo sapiens*.

a)

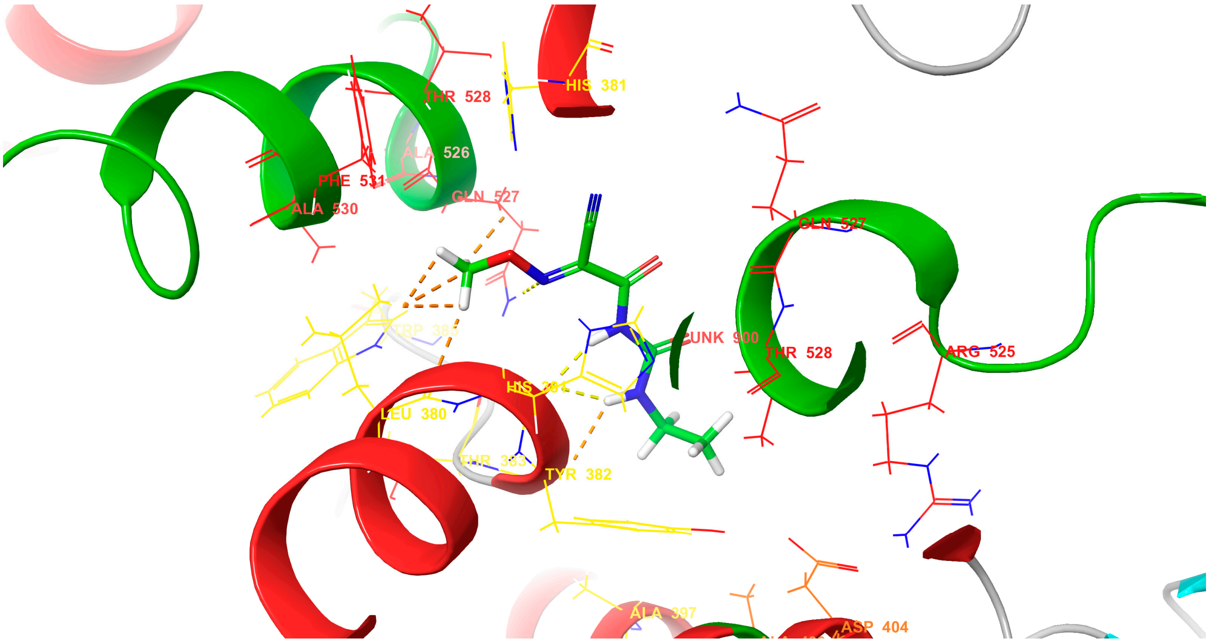

b)

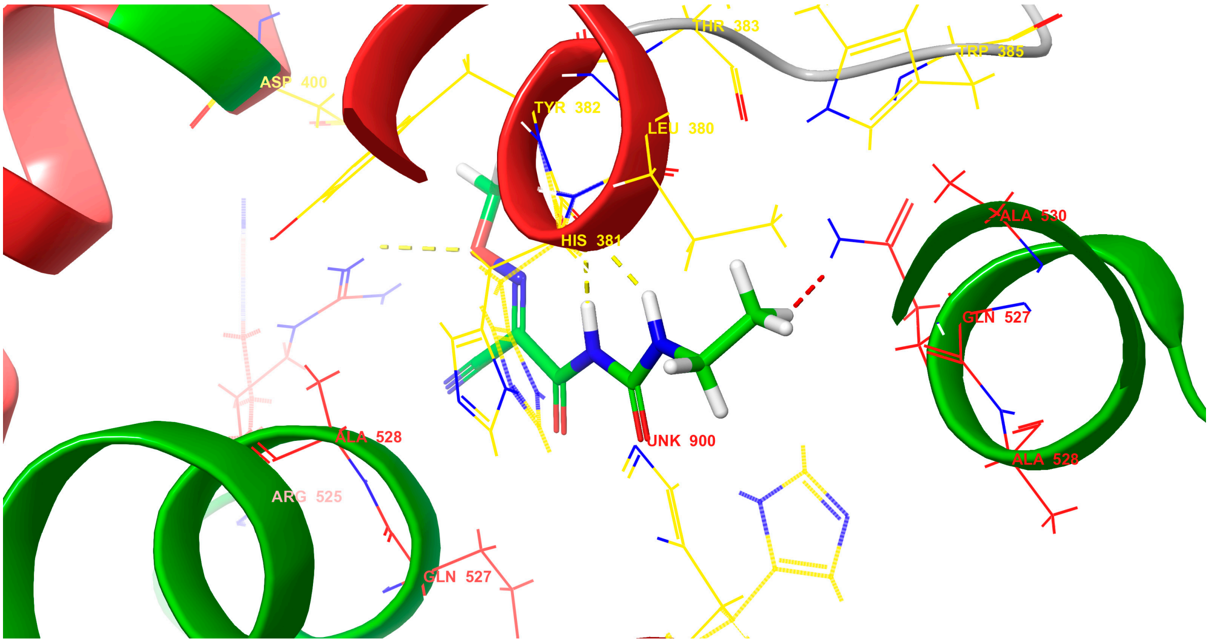

Figure S6. Graphic representations of docking cymoxanil into the structure of acetylcholine esterase from a) *Mus musculus*; b) *Homo sapiens*.

a)

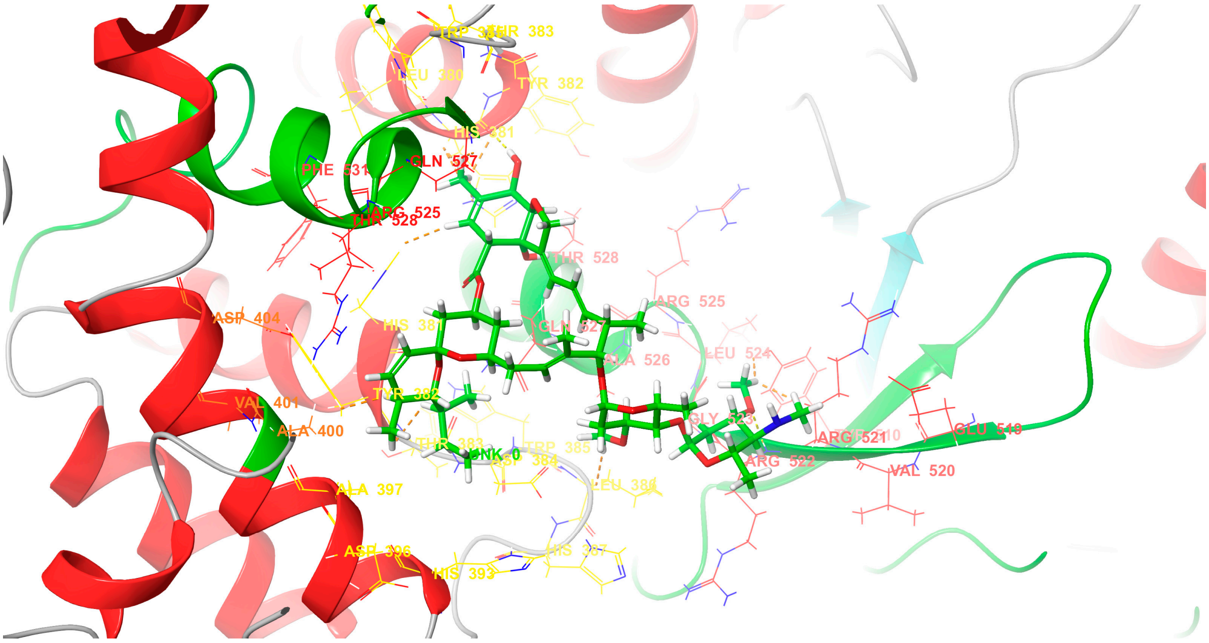

b)

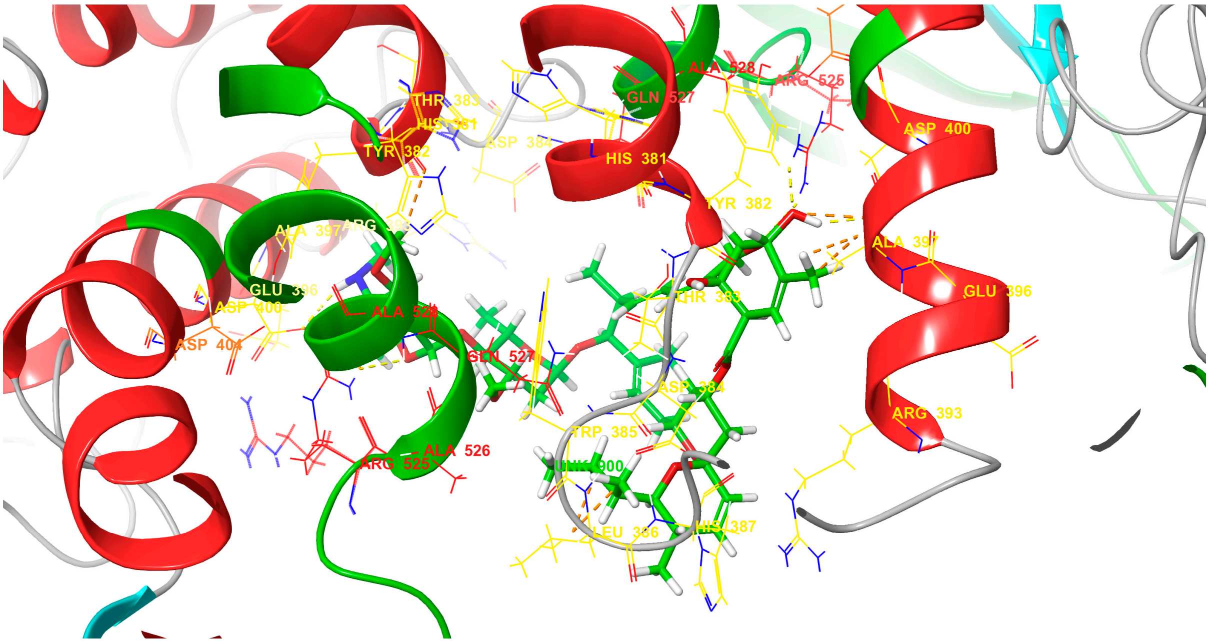

Figure S7. Graphic representations of docking emamectin into the structure of acetylcholine esterase from a) *Mus musculus*; b) *Homo sapiens*.

a)

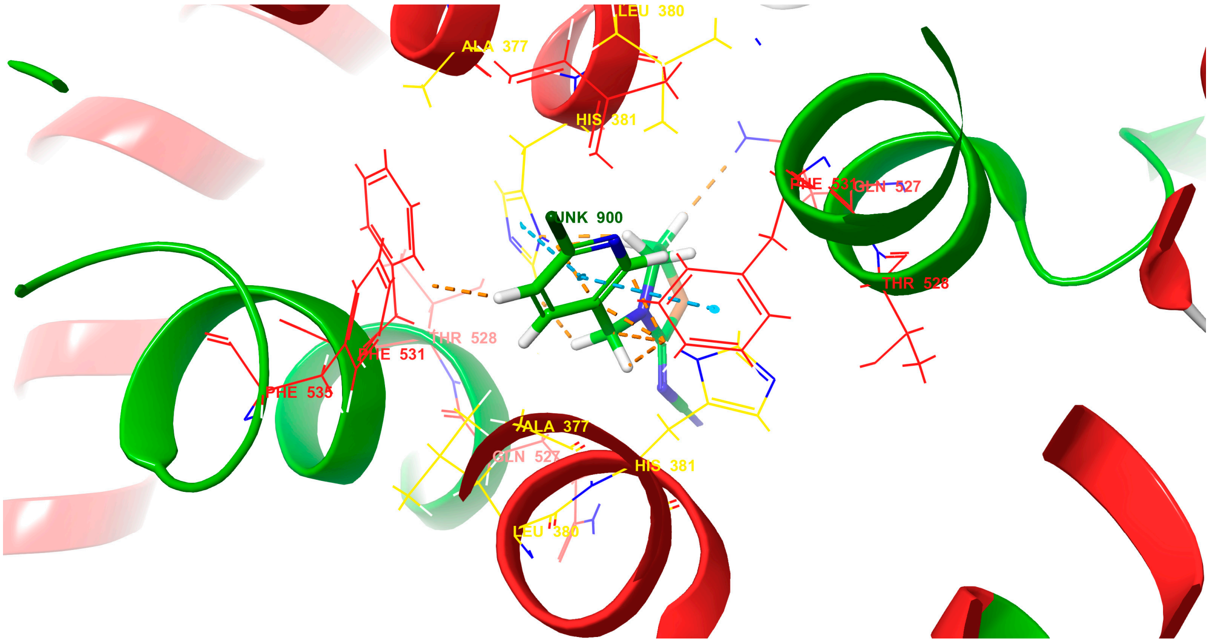

b)

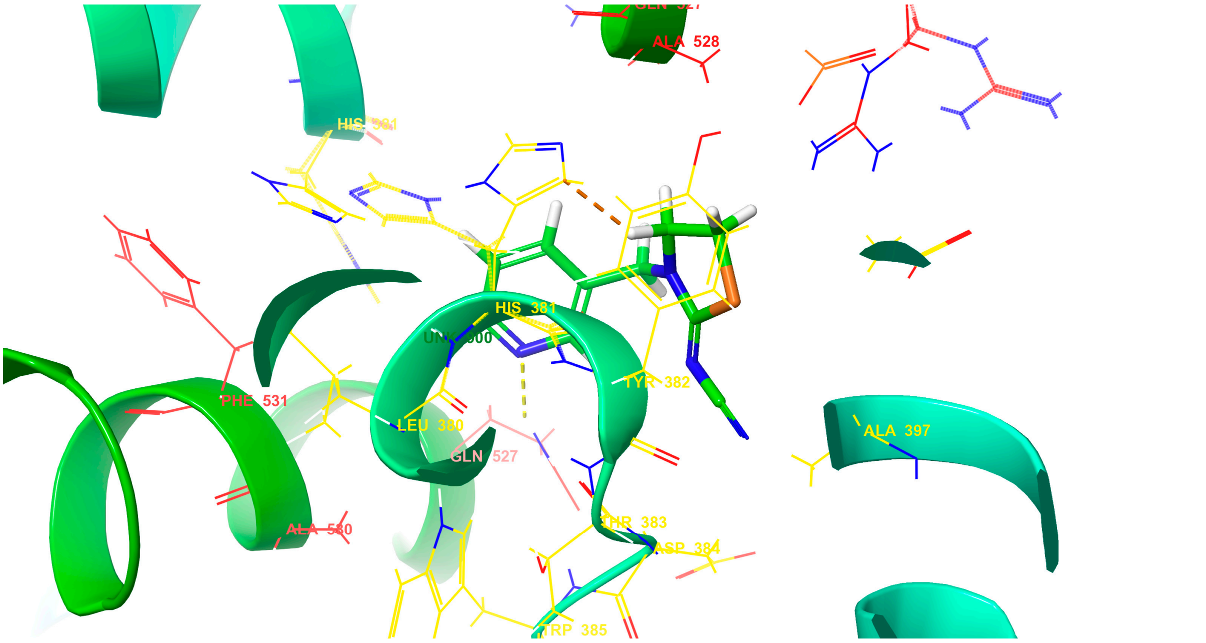

Figure S8. Graphic representations of docking thiacloprid into the structure of acetylcholine esterase from a) *Mus musculus*; b) *Homo sapiens*.

a)

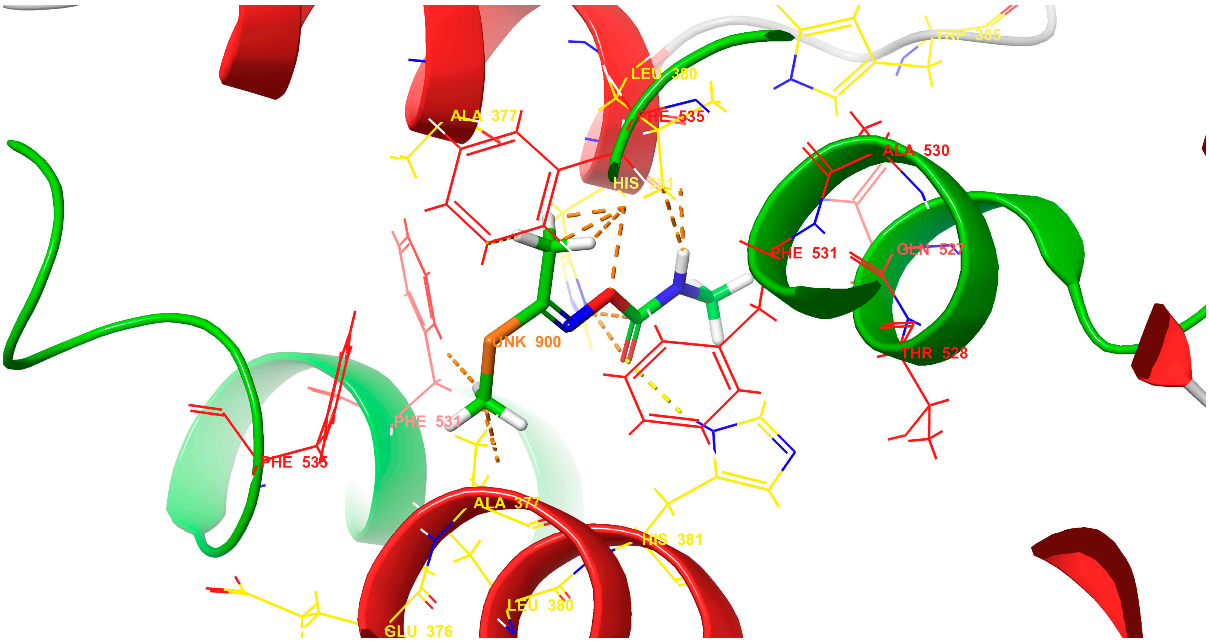

b)

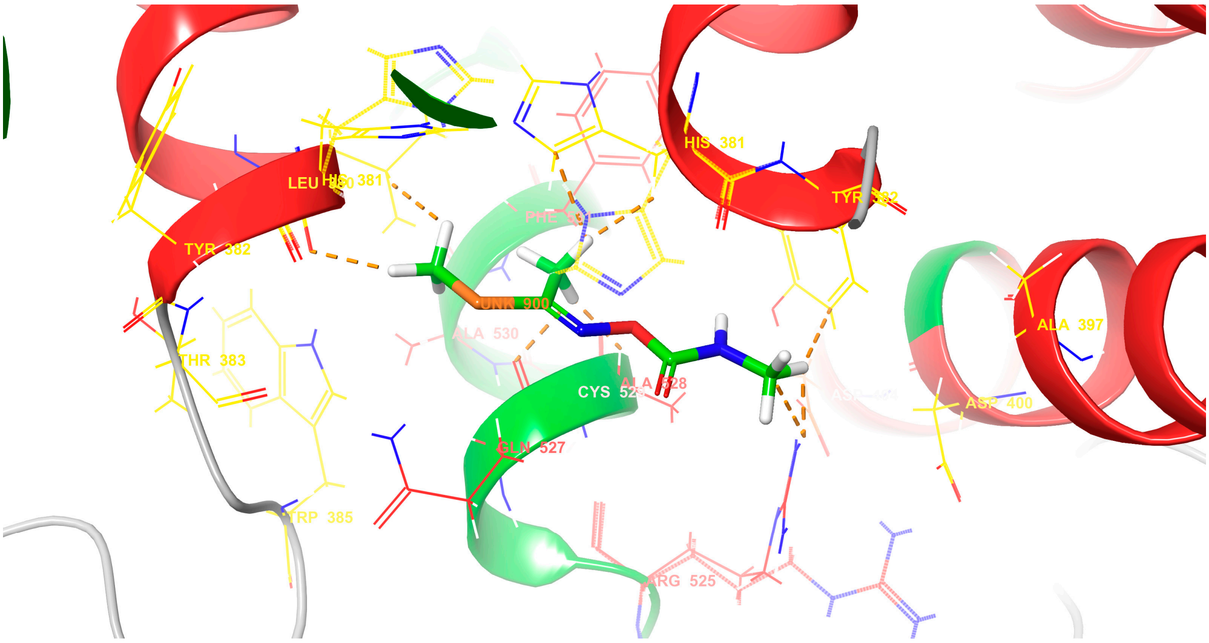

Figure S9. Graphic representations of docking methomyl into the structure of acetylcholine esterase from a) *Mus musculus*; b) *Homo sapiens*.

a)

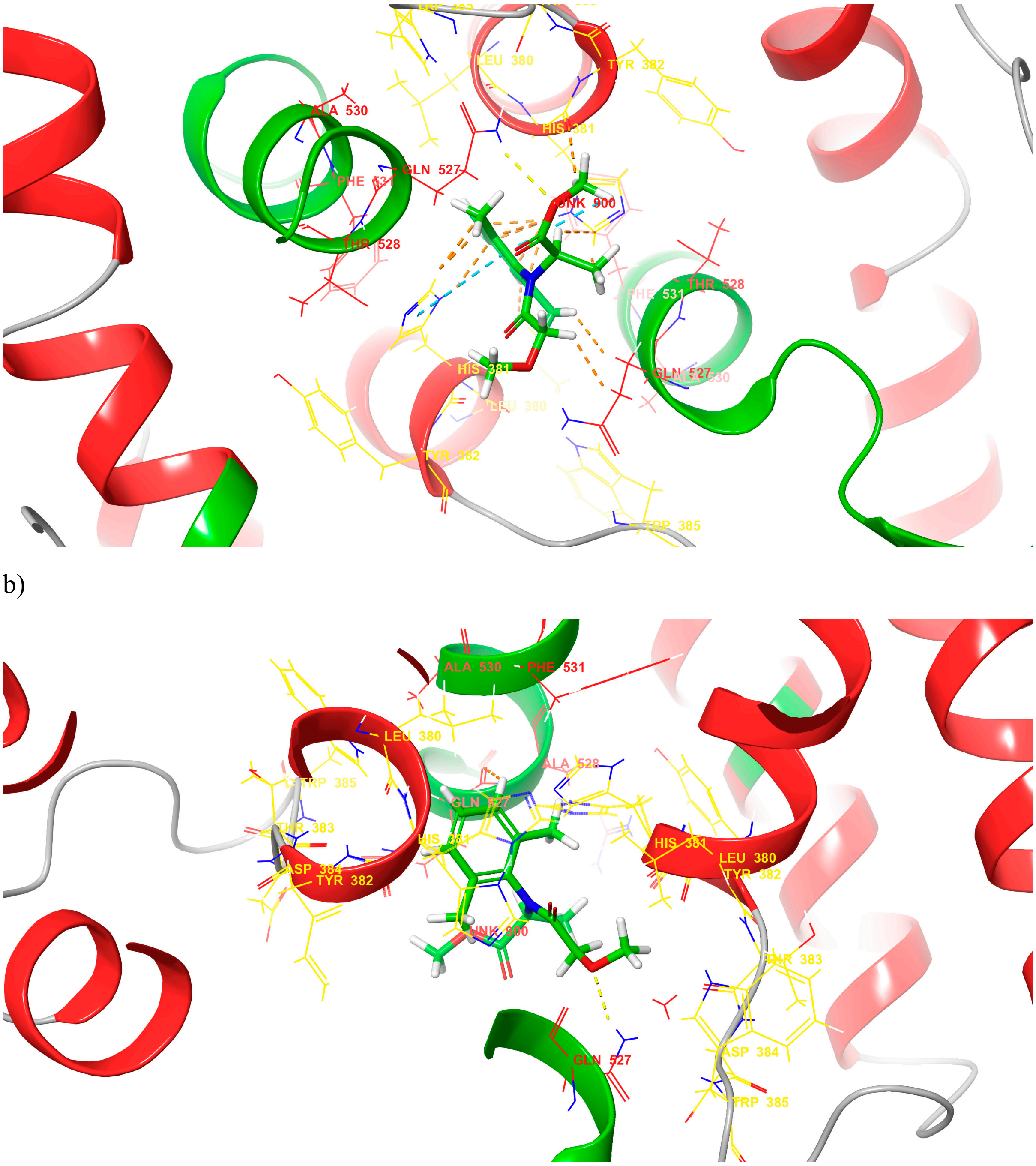

b)

Figure S10. Graphic representations of docking metalaxyl into the structure of acetylcholine esterase from a) *Mus musculus*; b) *Homo sapiens*.

a)

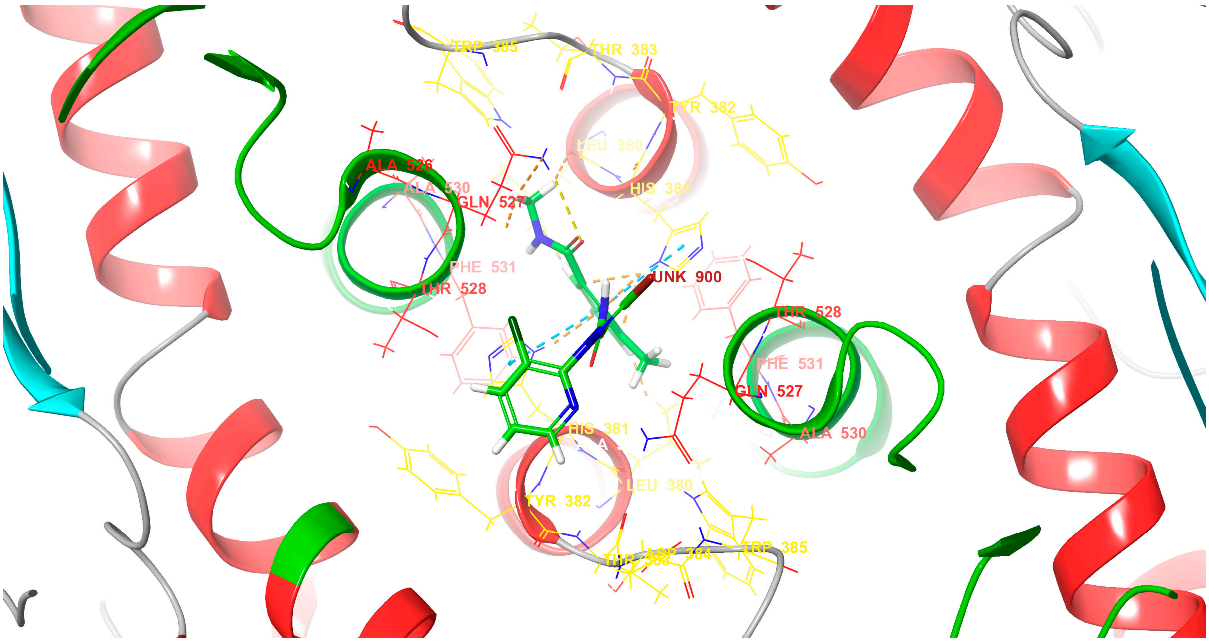

b)

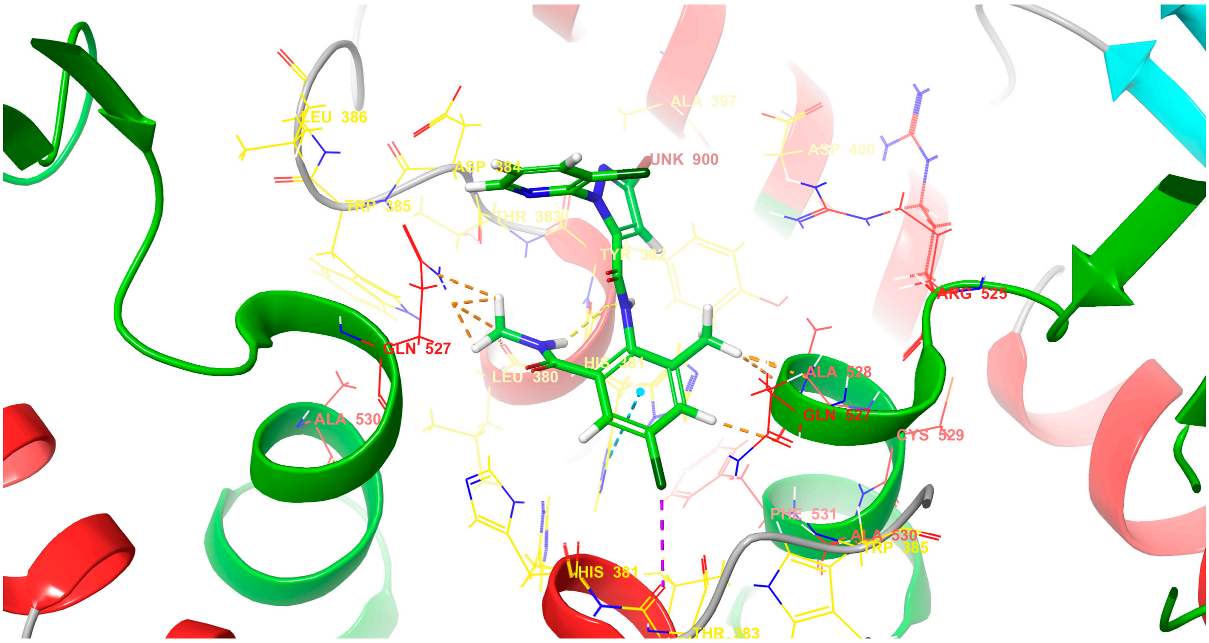

Figure S11. Graphic representations of docking chlorantraniliprole into the structure of acetylcholine esterase from a) *Mus musculus*; b) *Homo sapiens*.
